# Development of a Low Bias Method for Characterizing Viral Populations Using Next Generation Sequencing Technology

**DOI:** 10.1371/journal.pone.0013564

**Published:** 2010-10-22

**Authors:** Stephanie M. Willerth, Hélder A. M. Pedro, Lior Pachter, Laurent M. Humeau, Adam P. Arkin, David V. Schaffer

**Affiliations:** 1 Department of Chemical Engineering and the Helen Wills Neuroscience Institute, University of California, Berkeley, California, United States of America; 2 Department of Bioengineering, University of California, Berkeley, California, United States of America; 3 Department of Mathematics and Molecular and Cell Biology, University of California, Berkeley, California, United States of America; 4 VIRxSYS Corporation, Gaithersburg, Maryland, United States of America; Institut Pasteur, France

## Abstract

**Background:**

With an estimated 38 million people worldwide currently infected with human immunodeficiency virus (HIV), and an additional 4.1 million people becoming infected each year, it is important to understand how this virus mutates and develops resistance in order to design successful therapies.

**Methodology/Principal Findings:**

We report a novel experimental method for amplifying full-length HIV genomes without the use of sequence-specific primers for high throughput DNA sequencing, followed by assembly of full length viral genome sequences from the resulting large dataset. Illumina was chosen for sequencing due to its ability to provide greater coverage of the HIV genome compared to prior methods, allowing for more comprehensive characterization of the heterogeneity present in the HIV samples analyzed. Our novel amplification method in combination with Illumina sequencing was used to analyze two HIV populations: a homogenous HIV population based on the canonical NL4-3 strain and a heterogeneous viral population obtained from a HIV patient's infected T cells. In addition, the resulting sequence was analyzed using a new computational approach to obtain a consensus sequence and several metrics of diversity.

**Significance:**

This study demonstrates how a lower bias amplification method in combination with next generation DNA sequencing provides in-depth, complete coverage of the HIV genome, enabling a stronger characterization of the quasispecies present in a clinically relevant HIV population as well as future study of how HIV mutates in response to a selective pressure.

## Introduction

The development of a variety of next generation sequencing technologies – including Roche/454, Illumina/Solexa, Applied Biosystems SOLiD, Helicos Heliscope, and Pacific Biosciences Single Molecule Real Time (SMRT) sequencing – has made high throughput DNA sequencing possible. Each of these next generation sequencing methods offers differences in read length, the number of reads obtained, and the intrinsic error rate, all of which should be taken into account when choosing a specific sequencing platform. While next generation sequencing has only became commercially available in the past several years, it is already making a huge impact in a variety of biological fields, for example in sequencing of mammalian genomes and transcriptomes, studying different aspects of plant biology by allowing in depth characterization of the variability in these populations, and investigating viral evolution [1], [2], [3], [4], [5].

However, utilizing next generation sequencing to perform such studies poses numerous challenges. For example, library generation requires large quantities of DNA (µg) as starting material, and the use of this technology has thus been limited to studying cells or organisms where larger quantities of DNA can be readily isolated or where polymerase chain reaction (PCR) is used to amplify small quantities of DNA into sufficient quantities for library preparation. However, PCR can introduce a layer of bias into a sample since only sequences that contain significant homology with the primers sequences will be amplified, which will in turn affect the resulting analysis of diversity present in the sample. This study attempts to address this issue by developing a low bias method for amplifying small amounts of RNA and DNA - such as those isolated from tissue, rare cells, or clinical samples of pathogens - into larger quantities needed for next generation sequencing The resulting DNA can then be processed into libraries compatible with all of the aforementioned sequencing platforms. A further challenge is posed by the specific sequencing technology employed. Read length, sequencing error, and the amount of sequence obtained all play a role in the estimation of sequence diversity and error, and methods to account for all of these are constantly evolving. Here we apply both a new nucleic acid amplification and computational analysis that addresses the challenge of aligning next generation sequence data to a poorly defined reference genome for characterizing viral quasispecies.

The coupled approach potentially enables the characterization of entire viral populations, particularly important when studying viruses with a high mutation rate such as human immunodeficiency virus (HIV). An estimated 38 million people worldwide are currently infected with HIV, with an additional 4.1 million people becoming infected each year [6]. HIV is a member of the lentivirus family that infects cells found in the human immune system, primarily CD4+ T cells, leading to acquired immune deficiency syndrome (AIDS). The HIV genome consists of two positive strands of RNA that encode the various structural, regulatory, and accessory proteins required for HIV genome processing, integration, and replication inside infected cells [7]. Upon entering the cell, the HIV genome is then processed into double stranded DNA (dsDNA) via the enzymatic activity of reverse transcriptase found in the viral particle, and this DNA then integrates into the genome of the host cell. At this juncture, the provirus will either remain latent, allowing the cell to continue its normal functions, or will more often initiate rapid replication, resulting in the production of large quantities of viral particles and cell death [8]. Newly infected individuals tend to have relatively homogenous viral populations; however, due the high rate of virus production coupled with the error prone nature of reverse transcription, chronically infected HIV+ patients possess a highly heterogeneous viral population [9]. Additionally, on a global scale HIV has diversified into subtypes that differ considerably in genetic sequence [10], where subtype B is the predominant version in the Americas and Europe. Recombination between subtypes can also occur to generate circulating recombinant forms (CRF) of HIV [11].

Several important efforts have successfully employed 454 sequencing to examine different aspects of HIV biology [12]. In the initial work in this area, Bushman and colleagues used 454 sequencing combined with DNA barcoding to identify and quantify the frequency of rare HIV drug resistance mutations in patient plasma samples [13]. Another study demonstrated the utility of 454 deep sequencing technology to identify minor sequence variants in selected pieces of the HIV genome, the reverse transcriptase and HIV-1 protease genes, amplified using HIV sequence-specific primers [14], and a follow up study developed an algorithm for reconstructing the viral population within the sequenced sample, providing a valuable tool for studying the evolutionary dynamics of a viral population [15]. Another study used 454 sequencing to examine how a specific region of HIV *env* varies between two distinct viral populations, those infecting monocytes vs. T-lymphocytes [16]. Furthermore, 454 sequencing was used to study how a specific region of the HIV envelope mutated in response to treatment with a CCR5 agonist and to detect HIV that utilizes the CXC receptor 4, allowing for the phenotyping of specific HIV strains [17], [18]. More recently, 454 sequencing was used to characterize a recombinant form of HIV between the B and F subtypes [19]. This method used an HIV-based primer set to obtain 500-fold coverage of ∼8400 bases of the 9700 total that comprise the HIV genome.

The unprecedented read depth obtained using next generation sequencing can thus provide a deeper understanding of the variability present in an HIV population compared to traditional sequencing and can be further used to understand the biology behind HIV infection. However, the studies to date have utilized PCR for sample preparation, which in addition to likely amplifying a subset of the viral quasispecies also enabled sequencing of only a portion of the HIV genome. Accordingly, we have worked to develop a low bias method that, to our knowledge, for the first time does not entail the use of viral sequence specific primers and enables next generation sequencing of the full HIV genome from a clinically relevant sample. In addition, the methodology could generally be applied to analyze any RNA-based virus without any prior sequence knowledge, which would enable rapid characterization of viral outbreaks such as influenza, West Nile, Ebola, and severe acute respiratory system (SARS) [20]. Furthermore, the approach can enable the characterization of full viral genomes over time, for example in response to therapeutic intervention, allowing for more effective and efficient design of treatments.

To date, only 454 next generation sequencing has been applied to HIV. While both 454 and Illumina can enable rapid sequencing of genomes and transcriptomes [1], there are two major differences between the two technologies: read number and length. 454 produces approximately 400,000 relatively long (100–400 nt) reads per run (for each quad of a plate), with the number of reads likely to increase somewhat with newer instruments. In contrast, Illumina generates 10 to 25 million reads that currently range from 36 to 72 bps in length per run (for each of 8 lanes on a slide), with the option for reading both “paired ends” of a fragment to double the sequence obtained from a run, and read length with existing instruments is likely to continue growing. For this study, we have chosen to use Illumina sequencing due to its lower cost and increased coverage compared to 454 sequencing (360 MB–2.2 GB vs. 40–160 MB of sequence per run). Although the shorter Illumina read length enables less analysis of mutation linkage in a genome, the sequence obtained per run is considerably larger and thus enables a deeper analysis of a DNA sample, though it does pose computational challenges that we also address in this work.

In this study, a homogenous control population of HIV consisting of the NL4-3 strain was used to develop low bias amplification, Illumina sequencing, and computational analysis, resulting in complete coverage of the entire HIV genome with on average >40,000 bases per position. A heterogeneous, clinically relevant population of HIV produced by CD4+ T cells taken from a patient enrolled in a HIV clinical trial [21] was then analyzed. After developing and implementing an iterative computational method to determine a consensus sequence for this population, nearly 20,000 bases per position were recovered. To quantify the sequence diversity present in each sample, the base substitution rate was calculated for each population, and as anticipated, the clinical sample had higher diversity than the clonal sample on average. This study thus describes new means for low bias dsDNA amplification and preparation for sequencing on any platform, including 454, ABI SOLiD, Heliscope and Pacific Biosciences SMRT, coupled with computational reassembly and analysis of full length viral genomes from clinical samples.

## Results

**Figure 1:**
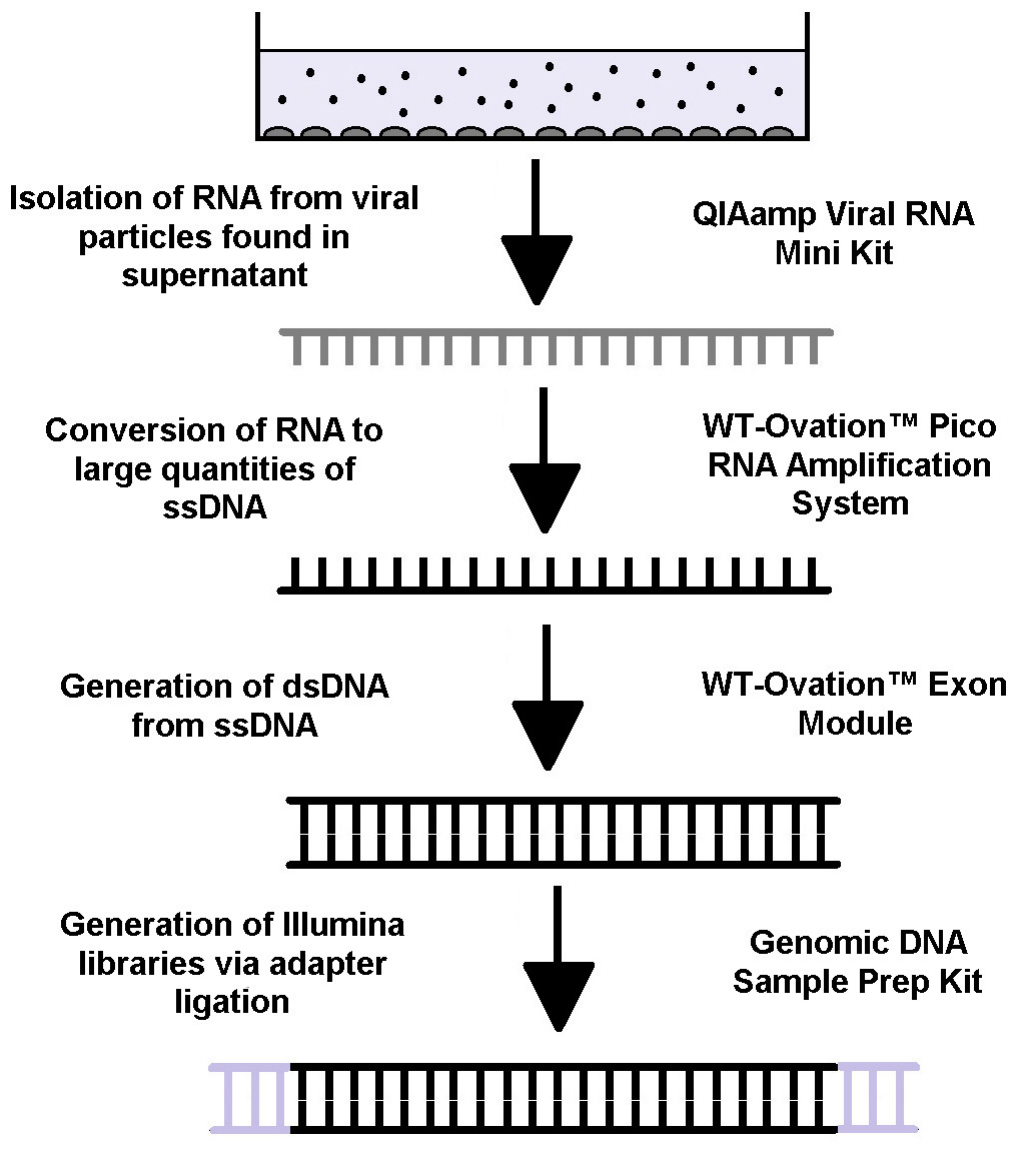
Flowchart of experimental methodology. Cell culture supernatants containing viral particles was collected from HIV infected cells. The RNA was extracted using the QIAamp Viral RNA Mini Kit and coverted into large quantities of single stranded DNA using the WT-Ovation Pico RNA Amplification System. The complementary strand for the ssDNA was then synthesized using the WT-Ovation Exon Module. The final step in the process involved using the Genomic DNA Sample Prep Kit to produce an Illumina library.

**Figure 2:**
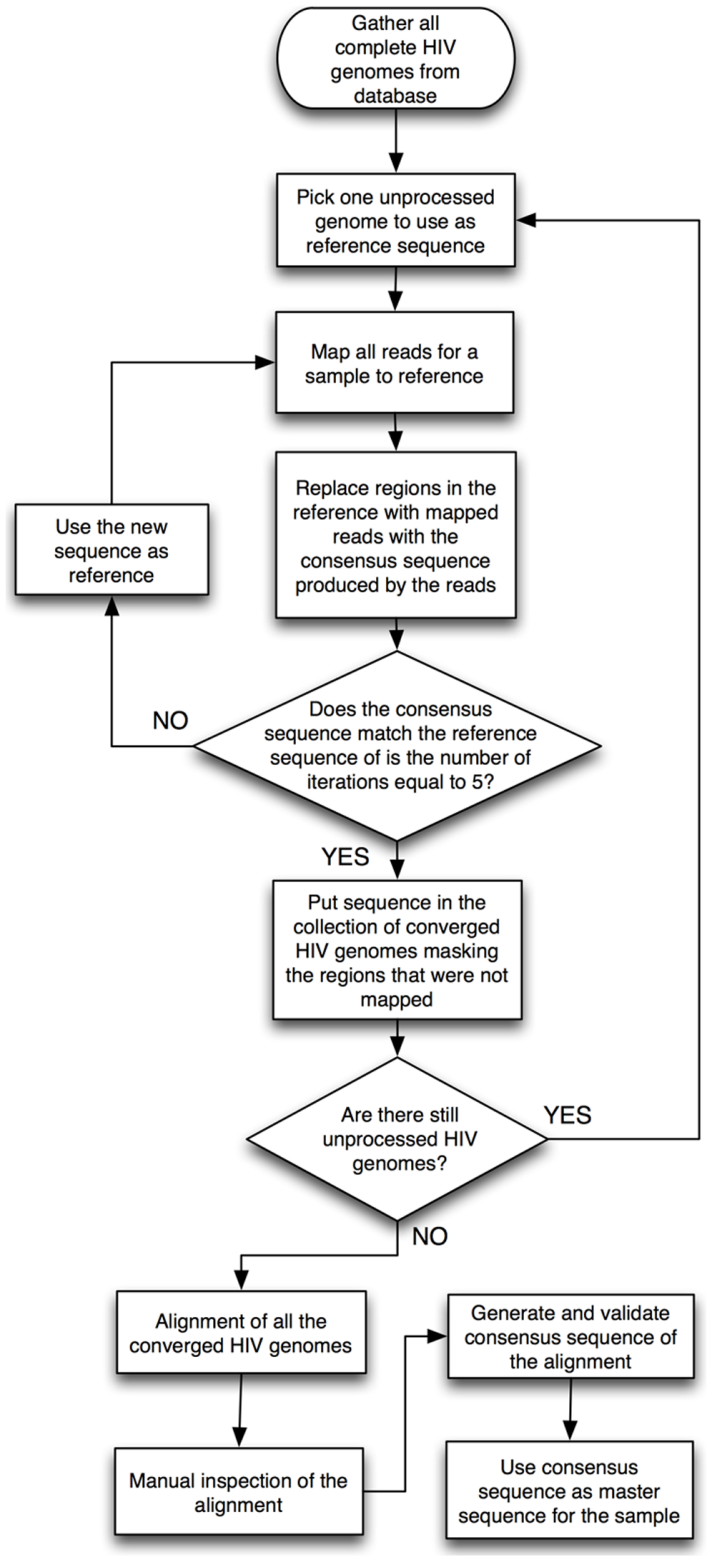
Flowchart of computational methodology. Once the millions of Illumina reads were obtained, they were then aligned against the 144 HIV-1 B genomes taken from the Los Alamos National Lab database. These alignments produced regions of consensus for each of these individual genomes which were then combined for the final alignment using MAFFT, which is a multiple sequence alignment program for nucleotide sequences.

The goal of this work is to develop a novel method for amplifying viral RNA into large quantities of DNA suitable for processing into Illumina (or other) libraries without relying on PCR and primers that assume specific sequences are present within the sample. By avoiding the use of virus-specific primers, one can reduce the bias associated with this step and potentially amplify the entire 9709 base pair HIV genome with unprecedented levels of coverage. Another challenge involved developing a novel computational method for reconstructing a “master sequence” for clinical patient samples with unknown sequences from the resulting reads obtained from the Illumina sequencing. To summarize these advances, the experimental portion of the workflow of our novel method is detailed in Figure 1, while the computational portion of the workflow is shown in Figure 2.

### Development of a novel method for generation of Illumina libraries from viral supernatants

Generation of an Illumina library requires large amounts of dsDNA (µg), while isolation of RNA from HIV supernatants tends to yield much lower quantites (ng). To address this issue, we developed a novel set of experimental methods to generate libraries from two different samples: a control homogeneous sample and a heterogeneous clinically relevant HIV sample. To generate the former, the canonical HIV strain NL4-3 [22] was produced using our previously published transfection method [23], and virus was collected after a propagation step. This sample served as a control sequence for this study, as the resulting viral population should contain essentially the same RNA sequence. The second sample, containing a clinically relevant HIV population, was derived from viral supernatant collected from an *in vitro* culture of CD4+ T lymphocytes obtained from a patient participating in a clinical trial. Specifically, VIRxSYS isolated CD4+ T lymphocytes from the peripheral blood of a HIV+ study subject as previously described [24], the T cells were later activated using immobilized CD3/28 beads [24], and viral supernatant was collected on day 9.

For both samples, we then used the QIAamp viral RNA Mini Kit to isolate viral RNA from cell culture supernatants, resulting in 30 µl of RNA with the concentration ranging between 1–5 ng/µl. This RNA was then converted into large quantities of single stranded DNA through the use of NuGEN WT-Ovation™ Pico RNA Amplification System, originally designed to process samples for microarray analysis, which yielded between 150–350 ng/µl of DNA with the total amount obtained ranging from 4.5 to 10.5 µg. This ssDNA was then converted into double stranded DNA using the WT-Ovation™ Exon module, originally designed for cDNA library synthesis, resulting in concentrations ranging between 300–500 ng/µl with the total amount obtained ranging from 9 to 15 µg. This dsDNA was then processed into an Illumina library using the procedure outlined in the Illumina Genomic DNA Sample Prep kit without performing the nebulization step, yielding libraries with a concentration between 10 and 20 ng/µl. More details on this experimental procedure can be found in the Methods sections and detailed protocols are given in Supplemental File S1.

#### Validation of Illumina Library Generation Protocol

To confirm that our method had worked for sequencing the entire HIV genome for the control NL4-3 sample, the corresponding library generated from that sample was then analyzed using paired end Illumina sequencing, resulting in 21,655,108 reads (Table 1). The NL4-3 sequence was then used as reference to map the Illumina reads from the same samples using the program Mapping and Assembly with Quality (MAQ), with the default parameters. An initial analysis revealed that the error rate varied as a function of position within the read (Methods, Supplemental Figure S1), and the last 6 nucleotides were thus trimmed. The trimmed paired-end reads were again aligned, and as detailed in Table 1, 71.75% of the reads mapped to the NL4-3 reference genome, with 59.15% mapping in pairs. 5.5% of the reads were possibly contaminated with adapter sequence and 1.72% of the reads mapped to the human genome.

**Table 1:** Composition of Illumina Reads Obtained.

Sample	Total Number of Reads	Number of reads mapping to the reference HIV genome	Number of reads mapping to the reference HIV genome in pairs	Number of reads with possible adapter contamination	Number of reads mapping to the human genome
**NL4-3 (homogenous)**	21,655,108	15,536,824 (71.7%)	12,808,867 (59.1%)	1,191,975 (5.5%)	371,752 (1.72%)
**Patient T cells (heterogeneous)**	14,632,646	6,869,780 (46.9%)	5,323,304 (36.4%)	1,052,221 (7.2%)	203,690 (1.39%)

**Figure 3:**
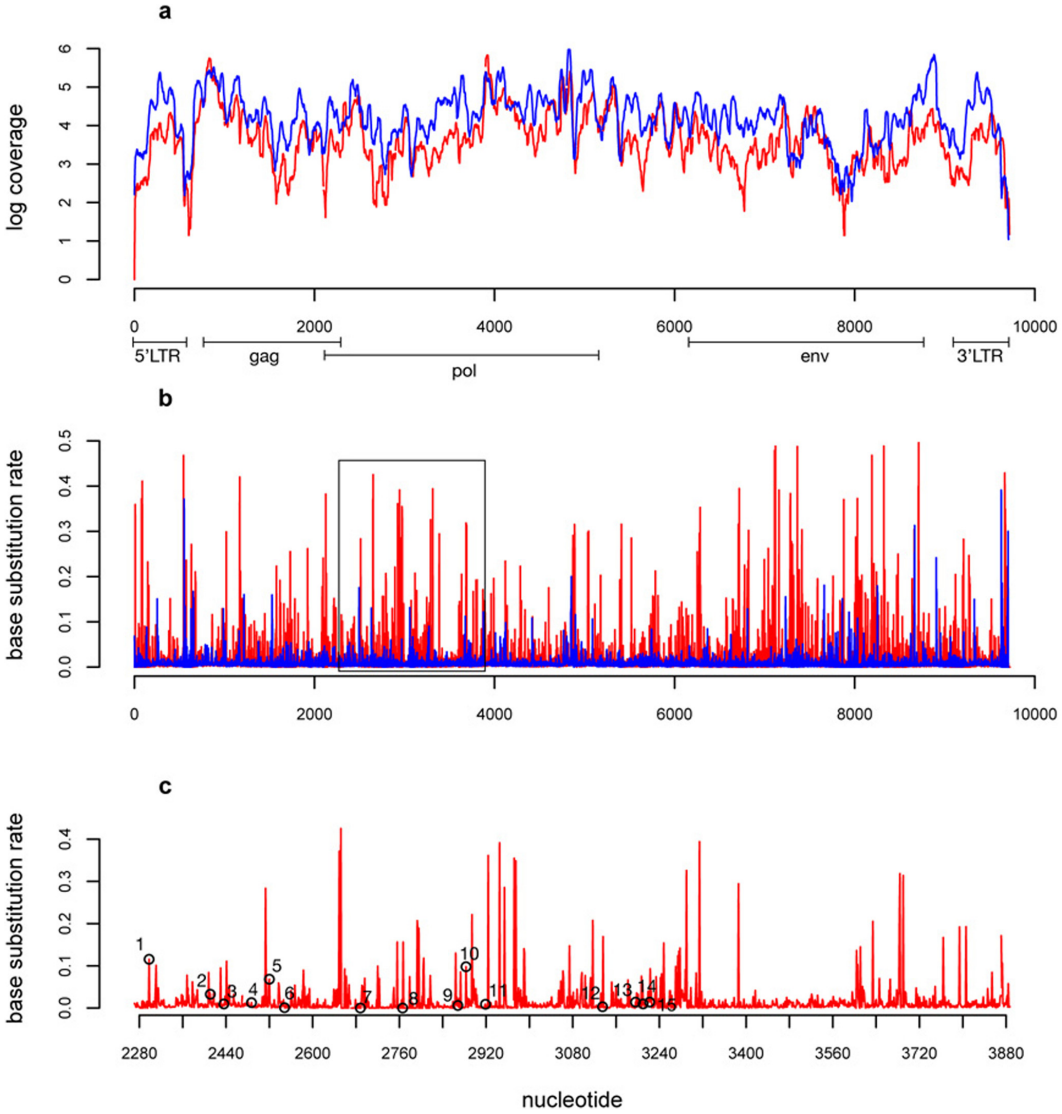
A) Coverage (log of read depth) of the HIV genome for both clonal and clinical samples. B) Base substitution rates plots for the same two samples. C) Magnified region of base substitution plot showing the location of the drug resistant mutations present in the clinical sample. The blue line corresponds to the coverage for the homogenous NL4-3 sample and the red line corresponds to the coverage of the heterogeneous clinically relevant HIV population. The average coverage was higher for the clonal sample than the clinical sample while the average base substitution rate was higher for the clinical sample. Key for drug resistant mutations in figure 3C (number- gene:mutation (basepair location)): 1 – PR:L10F (2298), 2 – PR:M46I (2411), 3- PR:I54V (2436), 4- PR:G73T (2487), 5- PR:I84V (2520), 6- PR:L90M (2548), 7- RT:M41L (2688), 8- RT:D67N (2766), 9- RT:K101H (2868), 10- RT:V106I (2883), 11- RT:V118I (2919), 12- RT:G190A (3135), 13- RT:L210W (3196), 14- RT:T215Y (3210), 15- RT:K219N (3222).

The entire NL4-3 reference genome was recovered with extremely high quality and depth of sequencing coverage, with an average of 44,116 bases aligning for each position. The maximum coverage was obtained at position 4832 of the reference genome in the *p31 int* region of the *pol* gene at a read depth of 951,240 (Figure 3A). To quantify the diversity present in the reads, the base substitution rate was calculated for each position, defined as the fraction of bases that do not map to the expected base for a given position (Figure 3B). The mean base substitution rate across the genome was 0.919% for this sample, which correlates well with the known sequencing error for the Illumina sequencing process [25]. The mean base substitution rates were also calculated for each of the genes of the HIV genome (*gag*, *pol*, *env*) as shown in Table 2. We did not identify any biologically relevant indels in this sample. While the *gag* and *pol* regions showed similar median base substitution rates, the rate of the *env* gene was statistically lower (Table 3).

**Table 2:** Mean base substitution rate for genes.

Sample	Mean base substitution rate for *gag* gene	Mean base substitution rate for *pol* gene	Mean base substitution rate for *env* gene
**NL4-3 (homogenous)**	0.902%	0.961%	0.853%
**Patient T cells (heterogeneous)**	1.459%	1.597%	1.707%

**Table 3:** Statistical Comparison of Base Substitution Rates in Different Genes**.

	*gag* clonal	*pol* clonal	*env* clonal	*gag* clinical	*pol* clinical
*pol* clonal	0.170				
*env* clonal	1.29E-14	2.20E-16			
*gag* clinical	3.75E-3	ND***	ND		
*pol* clinical	ND	0.0324	ND	0.0158	
*env clinical*	ND	ND	2.15E-4	5.01E-7	2.20E-16

**p values are given in this table.

***ND - Not determined, as the mean base substitution rates were statistically compared only for relevant pairs of samples.

### Reconstruction of a heterogeneous, clinically relevant HIV population

Having confirmed that our experimental method worked, we then focused on the data obtained from sequencing the “clinical sample,” which yielded over 14 million reads (Table 1). Unlike the NL4-3 sample, no known HIV genome was associated with the patient enrolled in the clinical trial. As a result, only a low number of these reads mapped to a single reference genome. To address this issue, we devised a novel methodology that would allow us to reconstruct a “consensus sequence” consisting of the most abundant sequence present for each region of the HIV genome for this specific patient. In particular, to fully utilize the amount of data obtained from our Illumina sequencing run as well as the sequence data present in the Los Alamos National Labs HIV Database, we developed an iterative strategy for enhanced mapping that utilized larger amounts of known sequence data for building HIV sequences (Figure 2). In this process, the reads were aligned against 414 known subtype B HIV-1 genomes from the Los Alamos National Laboratories using the iterative mapping method described in the Methods section, and the master reference sequence for this sample was constructed by combining regions of consensus where the Illumina reads mapped to each of these genomes. By taking the different regions of consensus that mapped to each of the 414 genomes, we were then able to generate our consensus sequence that could then be used as our reference sequence for mapping our Illumina reads. Our consensus sequence for this specific sample is given in Supplemental File S2.

After generating this consensus sequence for the clinical sample, we then were able to map the Illumina reads obtained against this new master sequence developed using the HIV genomes taken from the database, resulting in better coverage of the genome. On average, 17,148 bases mapped to each position of this novel reference HIV genome, corresponding to 46.95% of the total reads aligning to the genome along with 36.38% mapping as pairs, 7.2% being possibly contaminated with adapter sequence, and 1.32% mapping to the human genome. Supplemental Figure S2 shows the alignment of the clonal and clinical samples. The coverage as a function of position (Figure 3A) was maximal at position 3921, where the depth reached 676,897 reads. Interestingly, as observed for the clonal NL4-3 sample, this maximum occurs in the *pol* gene, within the region that encodes the p15 RNase protein. Using the novel consensus sequence, the base substitution rate for each position in the genome was again calculated (Figure 3B). The mean base substitution rate for the entire genome was 1.52%, significantly higher than the rate of the clonal sample (p = 1.58E-10) and the expected error rate for Illumina sequencing [25]. Similarly, higher base substitution rates were observed for individual genes in this sample when compared to the clonal population (Table 3). An interesting observation is that, while in the clonal sample the *env* gene is the gene with lowest mean variability (0.85%), in the clinical sample the *env* gene that shows the most variability (1.71%) compared to the other genes for this sample, consistent with *env* being the most variable region of the HIV genome [26].

Computational analysis for insertion-deletion (indel) determination and identification is still in a very early stage of development. Nevertheless, MAQ offers a very simple program that aims to recover indels from the reads that had to be aligned using the Smith-Waterman algorithm. This process is used when one of the paired end reads maps but its partner does not. This algorithm can then be applied to determine the spacing of the insertion or deletion required to map the latter. After filtering, we were able to find two biological interesting indels in our sample, which were mutations also found in the Los Alamos National Lab HIV database when queried using basic local alignment search tool (BLAST). One is a 6 bp deletion at position 6675, present in 2.44% of the reads that map in that site, and the other a 3 bp deletion at the position 7321, with a relative fraction of 36.1% (Table 4). Both of the mutations occurred in the *env* gene.

**Table 4:** Location and analysis of indels from clinical sample.

Location in genome	Length and indel sequence	Frequency (occurs in % of reads)
6675	-6:TAGAAA	2.44%
7321	-3:GAA	36.1%

We also scanned the sequence obtained against Stanford University HIV Drug Resistance Database [27] to determine whether this clinically relevant sample contained drug resistance mutations. The master sequence was confirmed by the database to contain the appropriate, biologically relevant HIV genes, and several drug resistant mutations were also identified. We found several of these mutations present in the *pol* gene – specifically in the protease and reverse transcription regions, , and these mutations are listed in Table 5 and shown in Figure 3C along with the associated base substitution rates.

**Table 5:** List of resistance mutation for the clinical sample*.

Known PR resistance mutations	M46I (2411), I54V (2436), I84V (2520), L90M (2548), L10F (2298), G73T (2487)
Known RT resistance mutations	M41L (2688), D67N (2766), V118I (2919), L210W (3196), T215Y (3210), K219N (3222), K101H (2868), V106I (2883), G190A (3135)
Known IN resistance mutations	none

Mutations are listed by amino acid mutation within the specific gene with the basepair position being given in parentheses.

## Discussion

Numerous studies have previously used 454 next generation DNA sequencing to analyze different aspects of HIV biology [13], [14], [16], [17], [18], [19], [28], [29], [30]. However, these strong studies did reveal some limitations of the underlying methodology – the use of HIV specific primers that introduced a level of bias into the sequencing data and the difficulty associated with amplifying the entire 10,000 nucleotide HIV genome for analysis. Although it can sometimes be useful to enrich for a target sequence when studying larger, eurkaryotic genomes where coverage is otherwise limiting [31], [32], in the case of HIV, it would be advantageous to sequence the entire genome to gain insights into the composition of complex viral populations and investigate the dynamics of their evolution. The method detailed in our work avoids the use of any HIV sequence specific primers by using a combination of oligodT and random hexamers that prime throughout the genome. Importantly, this method combined with Illumina sequencing yielded full coverage of the HIV genome with unprecedented levels (>17,000×) compared to prior studies.

To analyze the extent of coverage of both a simple and a more complex sample, we sequenced a control viral sample, the canonical NL4-3 strain, as well as a clinical sample consisting of a heterogeneous HIV population. For the latter, we developed new computational methods for constructing a consensus sequence and characterizing the diversity of such populations. In both cases, the entire HIV genome was recovered with unprecedented levels of coverage (number of mapped bases at a position in the genome) compared to previous reports. Interestingly, the degree of coverage varied across the genome, though the shape of coverage distribution curves was similar for both samples (Figure 3), indicating that some bias may have been introduced by the use of random hexamer primers and the mapping process to result in different extents of very deep coverage. As expected, the coverage of the clinical sample was lower than the clonal sample. This difference can be attributed to a number of possible explanations. First, certain reads obtained from the clinical sample will not map to any of the known genomes present in the database due to the diversity of the HIV population. Additionally, after generating the consensus sequence, there will be reads that will not map to this sequence because they are too divergent from the consensus “master” sequence despite previously mapping to some of the individual genomes in the database.

As an initial measure of diversity, we calculated the base substitution rates – defined as the fractions of bases that do not map to the expected base for a given position – for both samples, and the heterogeneous, clinically relevant HIV population was statistically more diverse. As shown in Tables 2 and 3, the median base substitution rate was significantly higher for all genes in the clinical patient compared to the homogenous clonal sample. Specifically, the base substitution rate for the *env* gene, the most variable region of the HIV genome [26], was the highest for the clinical sample, compared to 0.853% for the clonal sample. Additionally, in the clinical patient sample we observed two biologically relevant indels. Further analysis of this rich data set could yield more insight in the considerable sequence diversity of different regions of the HIV due to the levels of coverage. For instance, we were able to characterize the drug resistant nature of our clinically relevant HIV population (Table 5), and this information was consistent with the criteria for enrolling in the clinical trial (i.e. the patient had failed at least 1 regimen of HAART). Additionally, the generation of a consensus sequence for a patient serves as a valuable resource when observing how populations of HIV mutate over time.

One of the main limitations of using Illumina sequencing arises when attempting to determine whether a base pair that does not match the reference sequence is due to sequencing error or viral mutation. A potential method for distinguishing the difference between these two cases is to set a probability threshold based on the location of the mismatch in the Illumina read, as the sequencing error varies as a function of position within the read (Supplemental Figure S1), and the frequency with which a given mismatch is observed, where its appearance in numerous reads would indicate that it reflects true viral diversity. For the clonal sample, the error rate observed correlated well with previously reported Illumina error rates [25]. In the case of the clinical sample, however, it is more challenging to determine when a given mismatch was due to the sequencing method or to viral diversity. Additionally, it is possible that the mapping process used to establish a consensus sequence also introducfes a layer of error when assembling the consensus sequence. Therefore, while the novel iteration approach enabled the generation of a consensus sequence from >10 million short reads, we will explore this method in depth in future studies to fully utilize the potential of Illumina sequencing for studying viruses. Another alternative for addressing these limitations is to use SOLiD sequencing, which sequences each read in both directions – allowing for determination of when sequencing error occurs.

The experimental and computational methods developed here could be harnessed to investigate numerous biological problems, including how populations of HIV mutate over time in response to selective pressures, such as HAART or antiviral genetic therapies. In addition, a therapeutic pressure can result in the emergence of resistance due to mutations distant from the target site [23], and the ability to rapidly gain deep coverage of the entire genome (Figure 3A) will thus generally aid studies of HIV evolution. An example of such a therapy is Lexgenleucel-T, a cell-based therapy consisting of a dose of 10 billion autologous CD4^+^ T lymphocytes transduced *ex vivo* with the HIV-1 based lentiviral vector VRX96™, which encodes a 937 bp antisense sequence against the *env* region of HIV. Its introduction into CD4+ T cells was shown to inhibit HIV replication, and vector-transduced T cells were subsequently tested in Phase I clinical trials [21], [24], [33]. This sequencing method could be used to garner insights into the efficacy of this and other potential anti-HIV therapies, and knowledge gained from studying HIV evolution over time could potentially be used to design more effective therapies.

More generally, these experimental and computational methods can be used to study any DNA or RNA based virus with a known sequence to allow for the alignment of the Illumina reads. In addition, these methods could potentially be used to study pathogens without a known sequence, though this application would most likely require the development of methods for *de novo* assembly of the resulting Illumina reads. Furthermore, these methods can be applied to a diverse array of biological problems. Previously, next generation sequencing required such large amounts of starting material that it was applied to organisms and cell lines where scale-up would not pose an issue. This work will assist investigation of genomes and transcriptomes of cells and organisms that are harder to culture and as a result generate smaller quantities of genetic material available, such as primary cells, stem cells, and other cell lines that are difficult to culture in large quantities. Multiplexing of such samples to enable analysis of multiple libraries mixed into the same sample for sequencing can also render the process more cost effective and allow for the analysis of more samples. The experimental methodology detailed in this paper can also be used to prepare large quantities of dsDNA for any “next generation” sequencing platform. For these various reasons, this novel methodology expands the potential impact of next generation sequencing technologies.

## Methods

### Generation of HIV samples and Isolation of RNA

Two different HIV samples were processed and sequenced using Illumina technology in this study: a control virus produced via a transfection process and clinical viral sample produced by T cells obtained from an HIV+ patient participating in a study. The former was the NL4-3 viral strain [22], produced using a previously published transfection method [23], [34], and represented a homogenous viral population in which the viral genomes within the population should be nearly identical in sequence. To generate this virus, 12 µg of the sLTR plasmid was linearized using the restriction enzyme *Eco* RI and rejoined using ligase to remove the bacterial sequence present in plasmid as previously described [23]. HEK293Ts obtained from the American Type Culture Collection (www.atcc.org) were then transfected with this viral genome along with helper plasmids (3.5 µg of pcDNA3 IVS VSV-G, 5 µg of pMDLg/pRRE, 1.5 µg of pRSV Rev and 2 µg of CLPIT Tat-mCherry) to enhance production efficiency. The virus was amplified on SupT1s for 6 days (corresponding to approximately 3 rounds of amplification) to further increase the viral titer. Viral titer, determined as described previously [23], was approximately 40,000 IU/mL after the three rounds of amplification.

The second HIV sample, collected from *in vitro* culture of CD4+ T lymphocytes obtained from a patient participating in the VRX96™ phase II clinical trial (NIH Clinical Trial: NCT00131560) held by the VIRxSYS corporation, was anticipated to contain a heterogeneous HIV population. Patients enrolled in this study had already failed at least one HAART regimen and gave consent to for their cells to be used for research purposes Specifically, the CD4+ T lymphocytes were isolated from the peripheral blood from an HIV+ study subject as previously described [24] and frozen as stocks. For this study, these cells were thawed and allowed to recover overnight. The next day (Day 1), these cells were activated using immobilized CD3/28 beads as previously described [24], and on Day 9 supernatants from these cultures were collected for RNA isolation. The sample analyzed contained 425 ng/mL of p24 protein as determined using a p24 enzyme linked immunosorption assay. For samples, viral RNA was isolated from 540 µl of supernatant using the QIAamp viral RNA mini kit per manufacturer's instructions (Qiagen) and eluted in 30 µl of buffer AVE. No DNAse treatment step was performed.

### Double stranded DNA Synthesis from Viral RNA

To perform the processing necessary to produce an Illumina library, the nanogram scale quantities of viral RNA obtained after RNA extraction needed to be converted into microgram quantities of double stranded DNA (dsDNA). To this end, the NuGEN WT-Ovation™ Pico RNA Amplification System, which is generally intended for microarray applications, was initially used to generate large amounts of single stranded DNA from a small initial quantity of RNA using a three step process following the manufacturer's instructions. For detailed methods, please see Supplemental File S1. First, reverse transcription using Qiagen Sensiscript RT (estimated error rate between 1 mutation every 1700 basepairs and 1 mutation every 30,000 basepairs) converted HIV's RNA genomes into single stranded DNA (ssDNA). This first strand DNA synthesis reaction was initiated by a chimeric DNA/RNA primer set that allowed for the binding of reverse transcriptase to the RNA molecule. The DNA portion of the primer consisted of either a random hexamer or a polyT region followed by a unique RNA sequence that labeled the 5′ prime end of newly synthesized DNA molecule.

Next, second strand DNA synthesis was performed to generate a complimentary strand to the ssDNA generated during the first step. The final step in the process was the SPIA™ amplification step where a linear amplification process was performed on the dsDNA produced, generating large quantities of single stranded complimentary DNA (cDNA). This process requires DNA polymerase with an estimated mean error rate of 1 mutation every 100,000 basepairs.

Finally, to convert the purified cDNA into dsDNA, the WT-Ovation™ Exon module was used, in which a combination of random primers and DNA polymerase was used to generate the sense strand of the cDNA. This process requires DNA polymerase with an estimated mean error rate of 1 mutation every 100,000 basepairs. The resulting dsDNA products were purified using the QIAquick PCR purification kit with the modified protocol described in Supplemental File S1. The concentration of the resulting dsDNA was quantified using the ND-1000 spectrophotometer (Nanodrop) and confirmed using agarose gel electrophoresis.

### Preparation of DNA libraries for Illumina sequencing

The resulting dsDNA amplicons were then processed into a library using the following procedure taken from the Genomic DNA Sample Prep Kit (Illumina). No nebulization step was required because the input amplicons already ranged in size from 300–600 bp, and a more detailed protocol can be found in Supplemental File S1.

The size distribution and concentration of the Illumina libraries were then assessed using the Bioanalyzer 2000 (Agilent) by the University of California-Berkeley Functional Genomics Laboratory. For the NL4-3 sample, the average fragment size was 181 bp long, with a size distribution ranging from 120–210 bp in length. For the clinical sample, the average fragment size was 151 bp long with a size distribution ranging from 100–200 bp in length. A small portion of each library was cloned into the pSC-B-amp/kan plasmid using the Stratclone Blunt PCR Cloning Kit (Stratagene), and the resulting plasmids were Sanger sequenced to confirm the presence of HIV genome in these libraries. The resulting libraries were then deep sequenced on an Illumina 1G Genome Analyzer (Illumina) with paired end capability in the Vincent J. Coates Genomics Sequencing Laboratory at University of California-Berkeley. Read length was 36 base pairs.

### Data Filtering and Alignment

The raw data obtained from the Illumina runs were then processed using the default settings for base calling to ensure read quality with reads containing more than two unidentifiable bases were discarded, producing usable sequence data in standard FASTQ format. The reads were then mapped against the HIV reference genome that best represented the sample from where they came from using MAQ 0.7.1 software (Li H: **Maq: Mapping and Assembly with Qualities**. [http://maq.sourceforge.net/index.shtml]) allowing up to 3 mismatches in the first 24 bp of the read. A key requirement is to determine which part of the read has the most quality to be considered for population studies. Due to the chemistry of the sequencing reaction, the bases sequenced at the end of a read tend to have higher error rates, resulting in mismatches, indels, or ambiguously identified bases (Ns). To address this issue, we computed the error for each sequencing cycle in the NL4-3 control sample as shown in Supplemental Figure S1. To minimize the sequencing error, the last 6 bp of the reads were trimmed before analysis, resulting in 30 bp paired-end reads. This process has been shown to significantly decrease the error rate per base pair when using short reads [35]. This process allowed us to limit the number of mismatches observed due to the Illumina process and focus on the actual mutations due to viral evolution.

In contrast to the homogeneous control population of NL4-3, in which the Illumina data were mapped directly to its known sequence using the MAQ program, a new approach that accounted for variation in the input sequences had to be developed to reconstruct the clinically relevant, heterogeneous HIV population from the Illumina data. That is, members of a viral quasispecies obtained from an HIV+ patient will be highly dissimilar from any given single HIV reference sequence due to insertions, deletions and polymorphisms. Moreover, the quasispecies complexity, combined with the very short length of the reads, make de novo assembly of the full genomes impractical.

The millions of sequence reads were aligned against 414 distinct HIV-1 subtype B genome entries in the public Los Alamos National Labs HIV database (www.hiv.lanl.gov), and the reads that aligned to each reference genome were then used to generate new consensus sequences. That is, in the regions where reads mapped, the resulting consensus sequence was inserted in place of the corresponding region in the Los Alamos reference genome. In regions with no mapped reads, the reference sequence remained the same. All sequence data were again aligned to these new consensus sequences, updated reference sequences were again created, etc. As a result of five such iterations, there was a significant improvement in the number of mapped reads and in the fraction of the genome covered. The resulting consensus sequences (one for each original Los Alamos reference genome) were aligned to generate a final master consensus sequence. With this method, we were able to reconstruct an almost complete representative HIV genome of the clinical sample.

It was not possible to recover a particular region inside the *env* gene using this method, and we thus used a PERL assembly script to fill this region using overlapping reads. The final sequence was verified by manual inspection comparing it with the mapped reads by checking that the aligned reads overlapped without discontinuities.

We then used this sequence as the reference for the subsequent mapping and base pair substitution rate analysis. For both samples, we calculated the read depth (number of reads that align to a given position in the consensus) and base substitution rate (fraction of bases that are different from the consensus sequence), averaged across the entire genome and for specific genes of interest. To identify indels, we used the Smith-Waterman alignment found in the MAQ program. When one of the paired-end reads mapped to the reference and the other did not, MAQ tries to align the read in the expected region (based on the distance) using the SW algorithm, allowing us to identify significant indels in our sample, in particular specifically recurrent indels that originate from population diversity and not sequencing error.

#### Statistical Analysis

Since the error rate fit a Poisson distribution, we chose to use the Wilcoxon statistical test to compare the median error rates between the clinical and clonal samples and between the different genes for each samples. The Wilcoxon statistical test was carried out using the R software package (http://www.r-project.org/).

## Supporting Information

Figure S1 Plot of base substitution rate for the clonal sample as a function of read position. As shown in the plot, the first 6 base pairs of the read contain the highest amount of error.
(0.49 MB TIF)

Figure S2 Alignment of the clonal and clinical samples.
(2.03 MB TIF)

File S1 Detailed protocols for reproducing methodology.
(0.03 MB DOC)

File S2 Master Sequence based on Illumina sequencing of the clinical sample.
(0.03 MB DOC)
