# Supplementary figures and images for: Development of a Low Bias Method for Characterizing Viral Populations Using Next Generation Sequencing Technology
Source: PLoS One. 2010 Oct 22;5(10):e13564. doi: 10.1371/journal.pone.0013564 (PMC2962647; doi:10.1371/journal.pone.0013564)

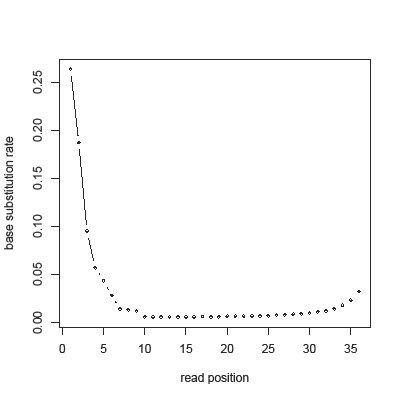

Supplement: Figure S1 — Plot of base substitution rate for the clonal sample as a function of read position. As shown in the plot, the first 6 base pairs of the read contain the highest amount of error. (0.49 MB TIF) [file pone.0013564.s001.tif]

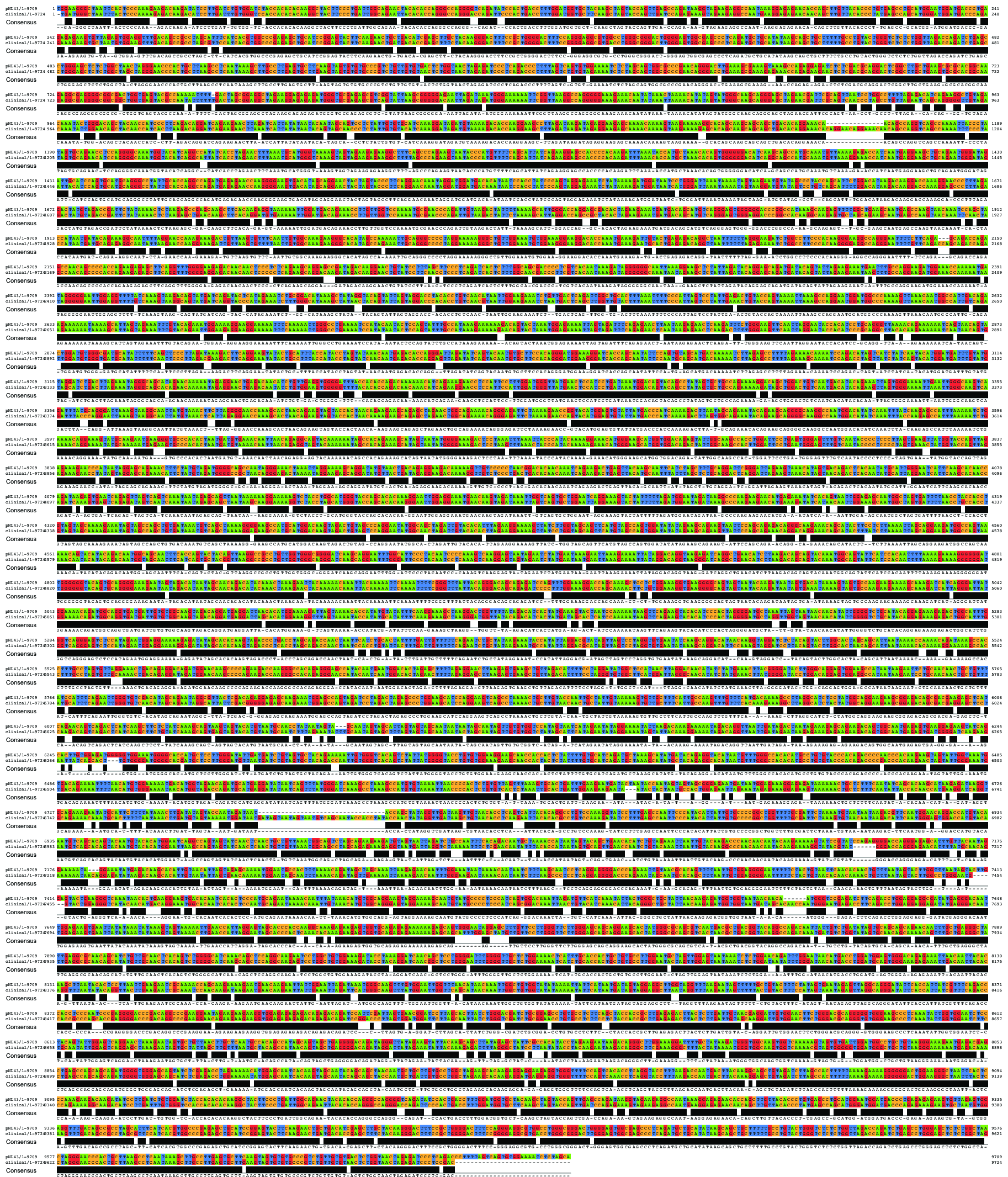

Supplement: Figure S2 — Alignment of the clonal and clinical samples. (2.03 MB TIF) [file pone.0013564.s002.tif]
