# Supplementary material for: Development of a Low Bias Method for Characterizing Viral Populations Using Next Generation Sequencing Technology
Source: PLoS One. 2010 Oct 22;5(10):e13564. doi: 10.1371/journal.pone.0013564 (PMC2962647; doi:10.1371/journal.pone.0013564)
Supplement: File S1 — Detailed protocols for reproducing methodology. (0.03 MB DOC) [file pone.0013564.s003.doc]

**Supplemental File One**

**Detailed Experimental Methods for Library Generation**

**WT-Ovation™ Pico Amplification System**

To amplify the small amount of viral RNA into large quantities of ssDNA, the WT-Ovation™ Pico Amplification System was used as follows. First, the First Strand cDNA Synthesis Protocol was followed according to the manufacturer’s instruction. Reverse transcription was used to convert HIV’s RNA genomes into single stranded DNA (ssDNA). This first strand DNA synthesis reaction was initiated by a chimeric DNA/RNA primer set that allowed for the binding of reverse transcriptase to the RNA molecule. The DNA portion of the primer consisted of either a random hexamer or a polyT region followed by a unique RNA sequence that labeled the 5’ prime end of newly synthesized DNA molecule.

Next, the Second Strand DNA Synthesis Protocol was performed according to the nanufacturer’s instructions to generate a complimentary strand to the ssDNA generated during the first step with the following modifications for the purification protocol. Agencourt© RNAClean© beads were used to purify the dsDNA. Before the purification process, 1 µL of 10 mg/mL yeast tRNA carrier (Ambion) was added, followed by 32 µL of beads. The mixture was then added to a 1.5 mL eppendorf tube, placed on a magnetic rack (Invitrogen), and allowed to incubate for 5 minutes to ensure binding of the beads to the wall of the tube. 45 µL of buffer was removed, and the beads were then washed 200 µL with freshly prepared 80% ethanol. This wash step was repeated twice more, and then the beads were allowed to air dry for 20 minutes.

The final step in the process was the SPIA™ amplification step where a linear amplification process was performed on the dsDNA produced , generating large quantities of single stranded complimentary DNA (cDNA). This protocol was performed per manufacturer’s instructions and the resulting reaction cleaned up using a modified protocol for the QIAquick PCR purification kit. The reaction contained the following reagents: DNA/RNA chimeric SPIA primer, DNA polymerase, and RNAse H to degrade the RNA portion of the primer and allow the binding of a new SPIA primer. The DNA polymerase was used to initiate DNA replication at the 3’ end of the DNA primer, which resulted in the displacement of the existing first strand. The process then repeated itself as the RNA portion of the SPIA primer was again removed by the activity of RNAse H followed by DNA synthesis. In this manner, large quantities of single stranded cDNA encoding the HIV genome were produced. The SPIA reaction was performed on the cDNA still bound to the magnetic beads by adding the SPIA primer mix along with the enzyme mix and appropriate buffer. The resulting reactions were then added to 1.5 mL eppendorff tubes and placed on the magnetic rack for 5 minutes to allow the beads to bind. The cleared supernatant containing the eluted cDNA products was collected, and the cDNA products were purified using the QIAquick PCR purification kit (Qiagen) with a slightly modified protocol. In particular, the two wash steps were performed using freshly prepared 80% ethanol. Also, to elute the DNA products from the column, 30 µL of nuclease-free distilled water was added to the center of the column and allowed to incubate for 5 minutes before the final spin.

**Generation of Illumina Libraries**

The resulting dsDNA amplicons were then processed into a library using the following procedure taken from the Genomic DNA Sample Prep Kit (Illumina) with enzymatic steps being carried out as specified bu the manufacturer. No nebulization step was required because the input amplicons already ranged in size from 300-600 bp The ends of the amplicons were repaired using the combination of T4 DNA polymerase and Klenow DNA polymerase to fill in the 5’ overhangs and remove the 3’ overhangs, and T4 polynucleotide kinase was used to phoshorylate the blunt ends of the amplicons at 20˚C for 30 minutes. The resulting products were then column purified using the QIAquick PCR purification kit. ‘A’ bases were then added to these amplicons using the polymerase activity of Klenow fragment (3’ to 5’ exo-) at 37˚C for 30 minutes, and the resulting products were purified using the MiniElute PCR purification kit (Qiagen). Adapter sequences were then ligated on to the amplicons at a 10:1 molar ratio during a 15 minute incubation at room temperature followed by purification using the QIAquick PCR purification kit. The resulting products were then run on a 2% agarose gel, and the 300-400 bp range containing the Illumina library was excised. The library was then isolated using Gel Extraction Kit (Qiagen). PCR was then used to further enrich and amplify the library. The program used started with 30 s at 98°C followed by 18 cycles of 10 s at 98°C, 30 s at 65°C 30 s at 72°C followed by a final extension step consisting of 5 minutes at 72°C. The follow primer set was used to generate paired end libraries: 5’ AATGATACGGCGACCACCGAGATCTACACTCTTTCCCTACACGACGCTCTTCCGATCT 3’ and 5’ CAAGCAGAAGACGGCATACGAGATCGGTCTCGGCATTCCTGCTGAACCGCTCTTCCGATCT 3’. The reaction was then cleaned up using the QIAquick PCR purification kit.
