# Supplementary material for: Development of a Low Bias Method for Characterizing Viral Populations Using Next Generation Sequencing Technology
Source: PLoS One. 2010 Oct 22;5(10):e13564. doi: 10.1371/journal.pone.0013564 (PMC2962647; doi:10.1371/journal.pone.0013564)
Supplement: File S2 — Master Sequence based on Illumina sequencing of the clinical sample. (0.03 MB DOC) [file pone.0013564.s004.doc]

**Supplementary File One: Sequence of the HIV genome of clinical sample**

ttgggagcagcaggaagcactatgggcgcagcgtcactaacgctgacggtacaggccagacaaatgatgtctggtatagtgcaacagcaaaacagtttgctgagggctattgaggcgcaacagcatatgttgcaactcacagtctggggcatcaagcagctccaggcaagagtcctggctgtggaaagatacctacaggatcaacggctcctgggaatttggggttgctctggaaaaatcatctgcaccactgctgtgccttggaatactagttggagtaataaatctctggataatatttgggataacatgacctggatgcagtgggaaagagaaattgacaattacacaggtttaatatacaggttacttgaagaatcgcaaatccaacaagataagaatgaacaagaattattgaaattagattcatgggcaagtttatggaattggttcagcataacaaattggctgtggtatataaaaatattcataatgatagtaggaggcttaataggtttaagaatagtttttactgtactttccatagtaaatagagttaggcagggatattcaccattatcgtttcagacccaccgcccagcaccgaggggacccgacaggcccgaaggaacagaagaagaaggtggagagagagacagagacagatccggacccttagtggatggattcttagcaattatctgggtcgatctgcggaacctgtgcctcttcctctaccaccgcttgagagacttactcttgattgtagcaaggattgtggaacttctgggacgcagggtgtgggaagccctcaaatattggtggaatcttctgcaatattggagtcaggagctaaagaatagtgctattagcttgtttaatgctacagccataacagtagctgaggggacagataggattatagaaatattacaaagagattttagggctatccttaatatacctacaagaataagacagggcttcgaaagggctttactataaaatgggtggcaagtggtcaaaacgtagtgggggtggatgggctgctgtaagggaaaaaatgagacaaactgagccagcagcagatggggtgggagcagcatctcgagacctggaaaaatatggagcaatcacaagtagcaatacagcagctaccaatgctgattgtgcctggctagaagcacaagaggatgaggaggtgggttttccagtcaaacctcaggtacctttaagaccaatgacttacaaaggagctgtagatcttagccactttttaaaagaaaaggggggactggaagggctaatttactcccagaaaaggcaagacatccttgatttgtgggtccaccacacacaaggctacttccctgattggcagaattacacaccagggccagggaccagattcccactgacctttggatggtgcttcaagctagtaccagttgatccagagaaagtagaagaggccaatgaaggagagaacaacagcttgttacaccctatgagccagcatgggatggatgacccagagaaagaagtgctaatgtggaagtttgacagccgcctagcgttccatcacgtggcccgagagctgcatccggagtacttcaagaactgatgacaccgagctttctacaagggactttccgctggggactttccggggaggcgtgacctgggcgggacttgggagtggcgagccctcagatgctgcatataagcagctgctttttgcctgtactgggtctctctggttagaccagatctgagcctgggagctctctggctagctagggaacccactgcttaagcctcaataaagcttgccttgagtgcttgaagtagtgtgtgcccgtctgttgtgtaactctggtaactagagatccctccgac
